# Clinical Relevance of Immune Checkpoints on Circulating Tumor Cells in Breast Cancer

**DOI:** 10.3390/cancers12020376

**Published:** 2020-02-06

**Authors:** Maria A. Papadaki, Anastasios V. Koutsopoulos, Panormitis G. Tsoulfas, Eleni Lagoudaki, Despoina Aggouraki, Alexia Monastirioti, Chara Koutoulaki, Christina A. Apostolopoulou, Aikaterini C. Merodoulaki, Chara Papadaki, Dimitrios Mavroudis, Sofia Agelaki

**Affiliations:** 1 Laboratory of Translational Oncology, School of Medicine, University of Crete, Heraklion, Vassilika Vouton, 71110 Crete, Greece; papadaki_maria1@yahoo.gr (M.A.P.); bio2241@edu.biology.uoc.gr (P.G.T.); daggouraki@yahoo.co.uk (D.A.); medp2011824@med.uoc.gr (A.M.); medp2011796@med.uoc.gr (C.K.); christinapostol@yahoo.gr (C.A.A.); bl01596@cc.uoi.gr (A.C.M.); chapapadak@uoc.gr (C.P.); mavrudis@med.uoc.gr (D.M.); 2 Department of Pathology, University General Hospital of Heraklion, Vassilika Vouton, 71110 Crete, Greece; akoutsop@med.uoc.gr (A.V.K.); medp1448@med.uoc.gr (E.L.); 3 Department of Medical Oncology, University General Hospital of Heraklion, Vassilika Vouton, 71110 Crete, Greece

**Keywords:** CD47, PD-L1, breast cancer, immune checkpoints, immune response, immune escape, liquid biopsy, CTCs, PBMCs, TILs

## Abstract

The role of CD47 and PD-L1 expression on circulating tumor cells (CTCs) remains unclear, and it is currently unknown whether their distribution varies between the blood and tumor tissue in breast cancer (BC). In this study, CD47 and PD-L1 expression was investigated a) on peripheral blood mononuclear cell (PBMC) cytospins from early (*n* = 100) and metastatic (*n* = 98) BC patients, by triple immunofluorescence for CD47/PD-L1/Cytokeratins, and b) on matched primary and/or metastatic tumor tissue from CTC-positive patients using immunohistochemistry. CD47+and/orPD-L1+ CTCs were detected in 11%, 16.9%, and 29.6% of early, recurrent, and de novo metastatic patients (*p* = 0.016). In metastatic disease, CD47^high^and/orPD-L1^high^ CTCs were associated with disease progression (*p* = 0.005) and shorter progression-free survival (PFS) (*p* = 0.010), and independently predicted for an increased risk of relapse (HR: 2.719; *p* = 0.008) and death (HR: 2.398; *p* = 0.034). PD-L1 expression rates differed between CTCs and tissue tumor cells and between peripheral blood mononuclear cells (PBMCs) and tumor-infiltrating lymphocytes (TILs) (positive concordance of 3.8% and 4%, respectively). CD47 expression also differed between CTCs and tumor cells (positive concordance of 11.5%). In conclusion, CTCs expressing CD47 and PD-L1 have independent poor prognostic implications in metastatic BC, indicating a potential role of innate and adaptive immune evasion mechanisms in their metastatic potential. The clinical value of the parallel assessment of the peripheral and local immune response merits further evaluation in BC.

## 1. Introduction

Although the development of novel therapeutic agents has significantly improved the survival rates of patients with breast cancer (BC), nearly 20–30% of those with early disease will relapse and ultimately die from their disease [1,2]. According to the theory of cancer immunosurveillance, the immune system has an important role in the elimination of cancer cells, as well as in the maintenance of tumors in a latent, non-clinically apparent state, for long periods of time [3,4]. Unfortunately, cancer cells exploit different mechanisms of immune evasion which enable them to grow and metastasize [3].

Immune cells of both the adaptive and innate immune system are involved in cancer immunosurveillance [5]. CD47 interacts with the myeloid inhibitory immunoreceptor signal-regulatory protein-α (SIRPα) to trigger a cascade of events that inhibit host cell phagocytosis, operating as a “do not eat me” signal [6]. CD47 is expressed on hematopoietic blood cells [7]; however, tumor cells frequently hijack its expression to escape macrophage scavenging [8]. CD47 expression on primary breast tumors has been associated with lymph node metastases and poor patient outcomes [9]. One of the mechanisms of adaptive immune evasion involves the inactivation and apoptosis of T lymphocytes expressing the programmed cell death receptor 1 (PD-1), through the interaction with the programmed cell death receptor ligand 1 (PD-L1) expressed on tumor cells [10]. In BC, modest and variable PD-L1 expression on tumor cells and/or tumor-infiltrating lymphocytes (TILs) has been described; however, divergent results exist on its prognostic significance [11]. Recent evidence suggests the oncogenic-driven co-expression of CD47 and PD-L1 on tumor cells [12], which, in co-operation, serve as two-step checkpoints in tumor immune evasion [13]. Therapeutic targeting of the interplay between cancer and immune cells has provided significant results in the treatment of patients with different solid tumors, especially using immune checkpoint inhibitors [14,15]. Even though BC is generally considered an immunologically “cold” tumor [16], accumulating evidence supports the role of different immunotherapy approaches in BC treatment [17].

Metastasis involves tumor cell detachment from the primary tumor and intravasation into the circulation, where they are identified as circulating tumor cells (CTCs) [18]. The presence of CTCs in the blood represents a crucial step in the metastatic process, as indicated by their prognostic value in various tumor types, including BC [19]. Importantly, the results of a recent meta-analysis strongly support the notion that CTC detection and enumeration represents a significant prognostic indicator in metastatic BC [20].

Immune cells have been demonstrated to expand the metastatic potential of CTCs within the bloodstream [21]. However, current research mostly focuses on the tumor immune crosstalk within the tumor microenvironment. Immune surveillance may differ between the primary tumor and metastatic sites [22,23]. Therefore, differential tumor-immune interactions may operate within the cancer tissue and peripheral blood. Indeed, CTCs have been shown to undergo phenotypic changes, such as epithelial-to-mesenchymal transition [24,25] that could contribute to the development of differential mechanisms of immune escape in CTCs compared to primary tumor cells [26,27]. In accordance, significant up-regulation of CD47 expression has been reported in CTCs compared to the corresponding tumor tissue in colorectal cancer [28]. In metastatic BC, CD47 is expressed in the majority of CTCs and is one of the putative markers identifying CTCs with a tumor-initiating capacity [8,9]. In a recent report, PD-L1-positive CTCs were detected in patients with hormone-receptor-positive/human epidermal growth factor receptor 2-negative (HR+/HER2−) metastatic BC [29]. However, the expression of PD-L1 or CD47 on CTCs has not been investigated in early BC, and no data exist on the clinical significance of their expression at the CTC level.

We hypothesize that the selection of less immunogenic CTC subsets may limit immune responses and favor metastatic progression. Conceivably, CTC characterization according to the expression of molecules involved in immune escape could provide significant information regarding the immune status of the tumor, which could be of relevance for the monitoring of disease evolution and the refinement of prognosis in patients with BC. We also hypothesize that the crosstalk of tumor and immune cells may differ between the blood and tumor tissue.

Based on the above, in the current study, we aimed to investigate the expression of CD47 and PD-L1 on single CTCs of patients with early and metastatic BC and to evaluate their prognostic role. We also performed a comparative analysis of the CD47 and PD-L1 status of tumor and immune cells in matched peripheral blood and primary and/or metastatic tumor tissue samples to investigate potential variability between the liquid and tumor biopsy. We demonstrate herein that the detection of CTCs expressing CD47 and PD-L1 holds an independent poor prognostic role in patients with metastatic BC. We also demonstrate a differential distribution of CD47 and PD-L1 expression in the blood and tumor tissue microenvironments, highlighting the importance of the peripheral immune response assessment in BC.

## 2. Results

### 2.1. CD47 and PD-L1 Expression in BC Cell Lines

The expression of CD47 and PD-L1 was evaluated in three human BC cell lines—SKBR-3, MCF-7, and MDA.MB.231—representing HER2-positive, luminal, and basal-like BC subtypes, respectively. A differential distribution of CD47 and PD-L1 was observed among the cell lines (Figure S1). High CD47 expression was detected in 55.7%, 85%, and 53.6% of SKBR3, MCF-7, and MDA.MB.231 cells, respectively. SKBR-3 and MCF-7 cell lines expressed low PD-L1 levels only, in 30.3% and 70.8% of cells, respectively, whereas the basal-like MDA.MB.231 cell line was the only one presenting high PD-L1 expression, in 82.6% of cells. Consequently, MDA.MB.231 cells were used as controls for CD47^high^ and PD-L1^high^ expression in the subsequent staining and characterization of patient samples. 

### 2.2. Patients

Patient and disease characteristics of early (*n* = 100) and metastatic (*n* = 98) BC patients are summarized in Table 1. At the time of analysis, 11 relapses and 11 deaths were recorded for early-stage patients (median DFI and OS, not reached). Metastatic patients had either recurrent (*n* = 71) or de novo metastatic (*n* = 27) disease, whereas at the time of analysis, 77 patients had relapsed (median PFS: 12.5 months (range, 9.9–15.1)) and 63 had died (median OS: 33.2 months (range, 27.3–39.1)).

### 2.3. CD47 and PD-L1 Expression on CTCs of Patients with Early, Recurrent, and de Novo Metastatic BC

Total CK+ CTCs were detected in 15% of early (median CTC nu: 1; range: 1–19) and 22.4% of metastatic (median CTC nu: 1; range: 1–12) patients. Specifically, 19.7% and 29.6% of patients with recurrent and de novo metastatic BC, respectively, were CTC-positive.

**Figure 1:**
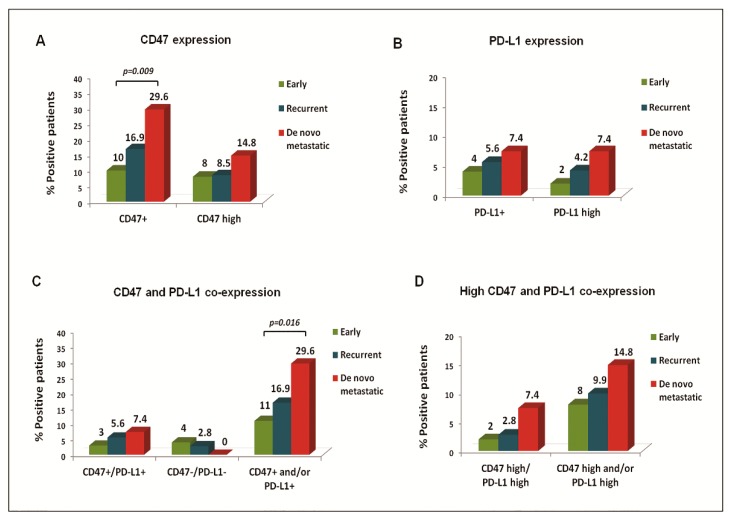
CD47 and PD-L1 expression rates on circulating tumor cells (CTCs) of BC patients. Frequency of distinct CTC subsets among patients with early, recurrent, and de novo metastatic BC. Percentage of patients with CTCs presenting (**A**) CD47 or CD47^high^ expression, (**B**) PD-L1 or PD-L1^high^ expression, (**C**) CD47 and PD-L1 co-expression, and (**D**) CD47^high^ and PD-L1^high^ co-expression.

CD47+ CTCs were evident in all settings; however, their frequency increased incrementally between patients with early, recurrent, and de novo metastatic BC (*p* = 0.036) and especially between early and de novo metastatic disease (*p* = 0.009). The detection rate of CD47^high^ CTCs was rather infrequent in all disease settings (Figure 1A). At the CTC level, CD47 was expressed in 83.8%, 91.3%, and 100% of total cells detected in early, recurrent, and de novo metastatic patients, respectively. 

PD-L1+ CTCs were identified in 4%, 5.6%, and 7.4% of patients with early, recurrent, and de novo metastatic disease (*p* = 0.669) and represented 21.6%, 21.7%, and 10% of total CTCs, respectively. The detection rate of PD-L1^high^ CTCs was 2%, 4.2%, and 7.4%, respectively (Figure 1B).

CTCs expressing at least one marker (CD47+and/orPD-L1+) were identified in 11%, 16.9%, and 29.6% of early, recurrent, and de novo metastatic patients, respectively (*p* = 0.059, early vs. de novo patients; *p* = 0.016) (Figure 1C). Moreover, the detection of CD47^high^and/orPD-L1^high^ CTCs numerically prevailed in de novo metastatic disease (Figure 1D), where all CD47+/PD-L1+ CTCs presented high expression levels of both markers.

CTC clusters were identified in two patients—one with early and one with de novo metastatic disease—who also harbored single CTCs. The patient with early BC had one single CD47^high^/PD-L1^neg^ CTC, and three clusters of the following phenotypes: CD47^high^/PD-L1^neg^ (*n* = 10 cells), CD47^high^/PD-L1^high^ (*n* = 4 cells), and CD47^low^/PD-L1^neg^ (*n* = 4 cells). Interestingly, the same phenotype was identified in all CTCs within each individual cluster. The metastatic patient harbored one single CD47^high^/PD-L1^high^ CTC and one cluster of two CTCs, both presenting CD47^high^/PD-L1^high^ expression.

**Figure 2:**
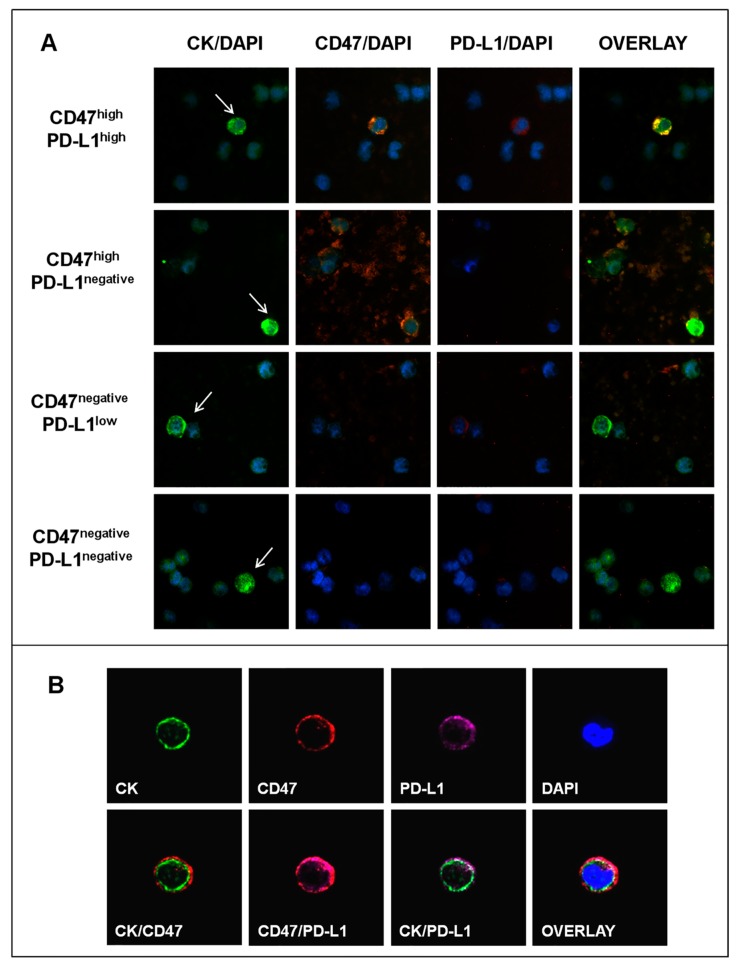
CD47 and PD-L1 expression on CTCs of BC patients. (**A**) Representative images of phenotypically-distinct CTC subsets according to CD47 and/or PD-L1 co-expression, Ariol microscopy (400X). Arrows indicate CTCs (CK+ cells) among peripheral blood mononuclear cells (PBMCs) (CK- cells). (**B**) A CTC (CK+ cell) positive for CD47 and PD-L1 expression, Confocal Laser Scanning Microscopy (CLSM) (600×). Distinct localization of the three molecules at the single CTC level.

Representative Ariol microscopy images of the distinct CTC subsets are depicted in Figure 2A. Further evaluation using confocal microscopy revealed a clustered membranous CD47 distribution in all CD47+ CTCs (Figure 2B), which is required for successful binding to SIRPα and triggering of the inhibitory signal [30]. Moreover, in accordance with the reported pattern of PD-L1 expression in BC cells [31], all PD-L1+ CTCs presented membranous PD-L1 localization alone or in combination with cytoplasmic staining (Figure 2B), whereas nuclear PD-L1 expression was not observed.

### 2.4. Clinical Relevance of CD47 and PD-L1 Expression on CTCs in Metastatic BC

#### 2.4.1. Correlation of CTC Subsets with Clinicopathological Parameters and Response to First-Line Treatment

The detection of total CTCs or distinct CTC subsets was not associated with age, menopausal status, performance status, the number of organs affected, or the site of metastases.

A differential distribution of CTCs was observed among patients with distinct BC subtypes (Table 2). CTCs with a positive or high expression of CD47 and PD-L1 significantly prevailed in patients with triple-negative, compared to HR+/HER2− and HER2+, disease. CD47^high^and/orPD-L1^high^ CTCs presented the most significant correlation to the triple-negative subtype (identified in 33.3%, 11.1%, and 0% of patients, respectively, *p* = 0.015) (Table 2).

Regarding best response to first-line treatment, CTCs were more frequently detected in patients with progressive disease (PD), compared to those with partial response (PR) or stable disease (SD) (44.4% vs. 16.7%; *p* = 0.011). Similarly, the detection of CTCs with a positive or high CD47 and PD-L1 expression was correlated to PD, with CD47^high^and/orPD-L1^high^ CTCs providing the strongest association with PD (27.8% vs. 5.6%; *p* = 0.005) (Table 3).

#### 2.4.2. Correlation of CTC Subsets with Survival Measures

**Figure 3:**
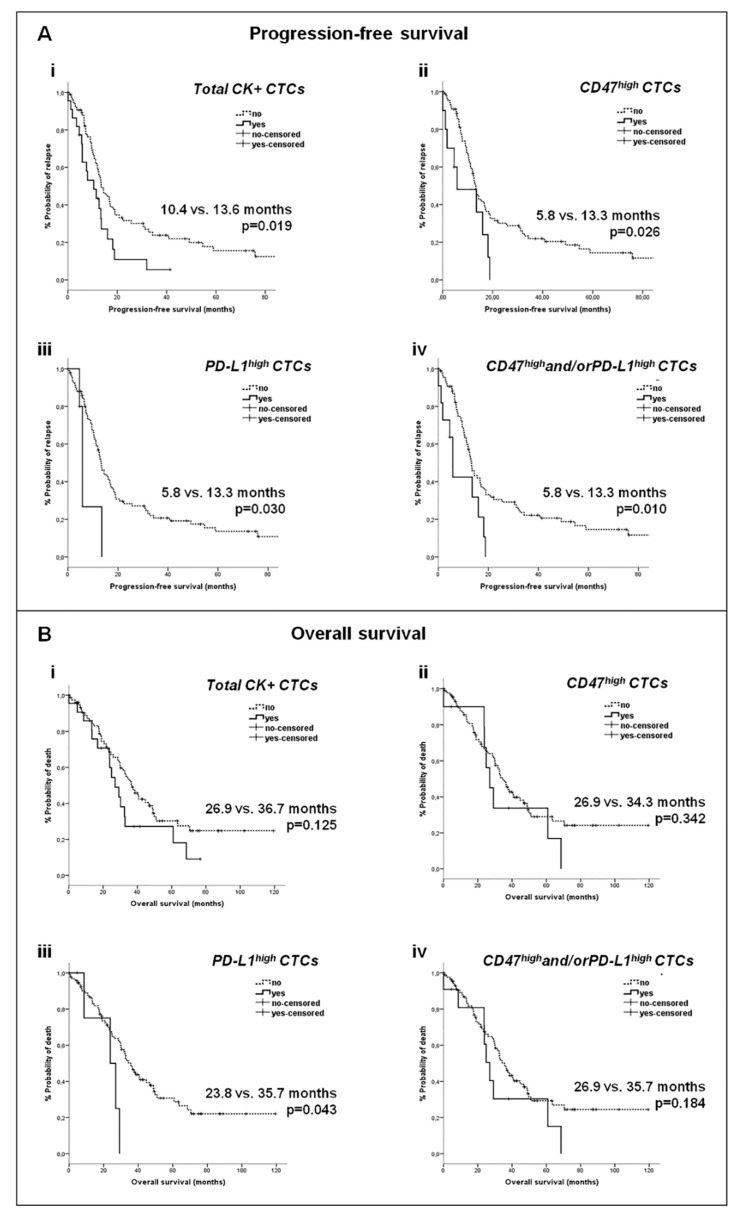
Survival analysis of patients with metastatic BC according to the detection and phenotype of CTCs. (**A**) Kaplan–Meier curves for progression-free survival (PFS) according to the detection of (**i**) total CTCs, (**ii**) CD47^high^ CTCs, (**iii**) PD-L1^high^ CTCs and (**iv**) CD47^high^and/orPD-L1^high^ CTCs, (**B**) Kaplan–Meier curves for overall survival (OS) according to the detection of (**i**) total CTCs, (**ii**) CD47^high^ CTCs, (**iii**) PD-L1^high^ CTCs and (**iv**) CD47^high^and/orPD-L1^high^ CTCs.

Τhe detection of total CTCs in patients with metastatic BC was associated with reduced progression-free survival (PFS) (*p* = 0.019) (Figure 3 (Ai)). Lower PFS rates were also recorded for patients bearing CD47^high^ CTCs (*p* = 0.026) or PD-L1^high^ CTCs (*p* = 0.030), and especially for those with CD47^high^and/orPD-L1^high^ CTCs (*p* = 0.010) (Figure 3 (Aii–iv)). Of note, there was no difference in PFS among patients with CD47^high^/PD-L1^high^ CTCs and those with CTCs presenting only CD47^high^ or PD-L1^high^ expression (*p* = 0.599) (Figure S2). Patients harboring PD-L1^high^ CTCs also had significantly shorter overall survival (OS) rates (*p* = 0.043) (Figure 3 (Biii)).

Univariate Cox-regression analysis revealed an increased risk of relapse for patients with CTCs (*p* = 0.021), CD47^high^ CTCs (*p* = 0.030), PD-L1^high^ CTCs (*p* = 0.039), or CD47^high^and/orPD-L1^high^ CTCs (*p* = 0.013), and for those with liver metastases (*p* = 0.042) (Table 4). In multivariate analysis, the detection of CD47^high^and/orPD-L1^high^ CTCs (*p* = 0.008) and the presence of liver metastases (*p* = 0.014) independently predicted for an increased risk of relapse (Table 4).

An increased risk of death was revealed among patients with recurrent BC (*p* = 0.045), liver metastases (*p* = 0.008), or with metastases in more than two organs (*p* = 0.005) (univariate Cox-regression analysis). In multivariate analysis, CD47^high^and/orPD-L1^high^ CTCs (*p* = 0.034), recurrent BC (*p* = 0.000), liver metastases (*p* = 0.015), or metastases in more than two systems (*p* = 0.000) were independent factors predicting for an increased risk of death (Table 4).

### 2.5. Clinical Relevance of CD47 and PD-L1 Expression on CTCs in Early BC

No association was observed between the detection of total CTCs or CTC subsets and patient and disease characteristics in early BC. Their detection was also not associated with survival measures in Kaplan–Meier analysis or with the risk of relapse and death in the Cox proportional hazards regression model, which could be related to the fact that the median DFS and OS were not reached at the time of analysis.

### 2.6. Comparative Analysis of CD47 and PD-L1 Expression on Tumor and Immune Cells in Matched Peripheral Blood and Tissue Samples

To investigate the distribution of CD47 and PD-L1 on tumor and immune cells within peripheral blood and the tumor microenvironment, we evaluated matched blood and primary and/or metastatic tissue samples from CTC-positive patients.

**Figure 4:**
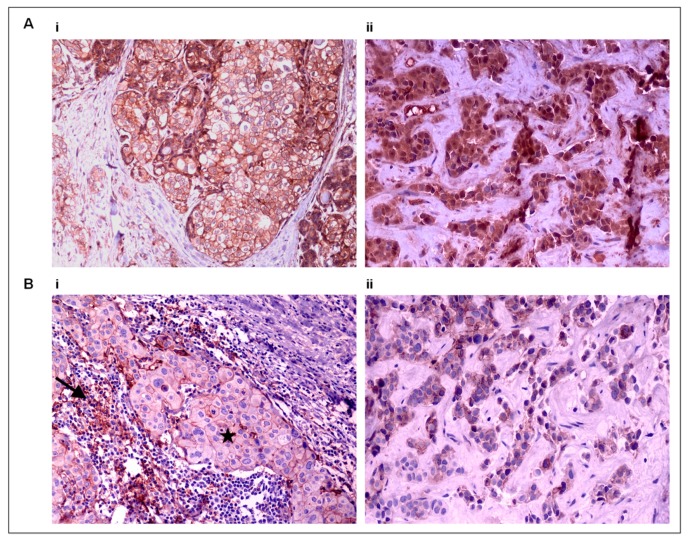
Tissue CD47 and PD-L1 expression by immunohistochemistry (IHC). (**A**) CD47 expression on tumor cells in (**i**) a primary BC tumor (×200) and (**ii**) the corresponding skin metastasis (×400), and (**B**) PD-L1 expression on tumor cells (star) and tumor-infiltrating lymphocytes (TILs) (arrow) in (**i**) a primary BC tumor (×200) and (**ii**) the corresponding liver metastasis (×400).

Representative images of CD47 and PD-L1 staining on tissue samples are shown in Figure 4. No differences in the staining patterns were observed between primary tumor and metastatic sites. 

The following comparisons were performed.

#### 2.6.1. CD47 and PD-L1 Expression on Tumor and Immune Cells within Peripheral Blood (CTC-Positive Patients, *n* = 36)

CD47+ CTCs and CD47^high^ CTCs were evident in 80.6% and 50% of patients, respectively, whereas all PBMCs exhibited the CD47^high^ phenotype.

PD-L1+ CTCs and PD-L1+ PBMCs were detected in 27.8% and 22.2% of patients, respectively (positivity concordance of 11.1%). PD-L1^high^ expression was more frequent among CTCs compared to PBMCs (in 19.4% and 5.6% of patients, respectively; positivity concordance of 2.8%) (Table 5). Interestingly, PD-L1+ PBMCs were more frequently identified in early disease than metastatic disease (40% vs. 9.5%; *p* = 0.046).

#### 2.6.2. PD-L1 Expression on Tumor and Immune Cells within Tumor Tissue

PD-L1+ tumor cells and PD-L1+ TILs were detected in 34.6% and 65.4% of primary tumors, respectively (positivity concordance of 34.6%), whereas PD-L1^high^ tumor cells and PD-L1^high^ TILs were evident in 11.5% and 42.3%, respectively (positivity concordance of 11.5%) (Table 5). A correlation between PD-L1 expression on tumor cells and TILs was recorded and of note, all primary tumors with PD-L1+ tumor cells also harbored PD-L1+ TILs (*p* = 0.009). Similarly, all primary tumors with PD-L1^high^ tumor cells also had PD-L1^high^ TILs (*p* = 0.063).

Regarding metastatic tumor samples, PD-L1+ tumor cells and PD-L1+ TILs were detected in 1/7 (14.3%) and 4/7 (57.1%) of tumors, respectively, with positivity concordance in 1/7 patients. In contrast, no PD-L1^high^ tumor cells were evident in metastatic tissues, whereas PD-L1^high^ TILs were identified in only one metastatic tissue (14.3%) (Table S1).

#### 2.6.3. CD47 and PD-L1 Expression on Tumor Cells in Matched Peripheral Blood and Tumor Tissue Samples

CD47+ primary tumor cells and CD47+ CTCs were evident in 88.5% and 84.6% of patients, respectively (positivity concordance of 76.9%) (Table 6). CD47^high^ expression was more frequently identified in CTCs compared to primary tumor cells (53.8% vs. 19.2% of patients, respectively; positivity concordance of 11.5%) (Table 6). Similarly, CD47 and in particular, CD47^high^ expression, was more frequently detected on CTCs compared to tumor cells in the corresponding metastatic sites (Table S1).

PD-L1+ primary tumor cells and PD-L1+ CTCs were similarly identified (34.6% and 30.8% of patients, respectively); however, positive concordance was observed in only 7.7% of cases. PD-L1^high^ expression was more frequent in CTCs compared to primary tumor cells (23.1% vs. 11.5% of patients, respectively; positive concordance of 3.8%) (Table 6) or tumor cells detected in metastatic sites (Table S1).

Interestingly, discordant results were also observed regarding the expression of CD47 and PD-L1 on tumor cells between primary and metastatic sites (Table S1).

#### 2.6.4. PD-L1 Expression on Immune Cells in Matched Peripheral Blood and Tumor Tissue Samples

PD-L1+ TILs and PD-L1+ PBMCs were detected in 64% and 20% of patients, respectively (positivity concordance of 16%). Accordingly, PD-L1^high^ expression was more frequently detected in TILs compared to PBMCs (44% vs. 8% of patients, respectively; concordance of 4%) (Table 6). Similarly, PD-L1 and PD-L1^high^ expression was more frequently observed in TILs within the metastatic tumor tissue compared to the corresponding PBMCs (Table S1).

## 3. Discussion

Here, we demonstrate, for the first time, that CD47 and PD-L1 are co-expressed on single CTCs in BC and that the incidence of CD47^high^ and/or PD-L1^high^ CTCs increases from early to de novo metastatic disease. Importantly, CD47^high^ and/or PD-L1^high^ CTCs independently predict for an increased risk of relapse and death in metastatic BC. Finally, CD47 and PD-L1 expression was characterized for the first time in matched tumor and immune cells within the blood and tumor tissue samples and discordance was shown in their distribution between the two compartments.

In this report, CD47 and PD-L1 were investigated in different stages of tumor progression: early, recurrent, and de novo metastatic BC. Interestingly, the incidence of CD47+ and PD-L1+ CTCs progressively increased from early to de novo metastatic disease. Moreover, it was shown, for the first time, that CD47 and PD-L1 are co-expressed on single CTCs and that double positive CTCs, as well as CD47^high^ and/or PD-L1^high^ CTCs, also prevail in de novo metastatic BC.

In line with our findings, Baccelli et al. provided direct evidence on the metastatic potential of CTCs expressing CD47, among other markers [8], and demonstrated CD47 expression in CTCs detected in a small cohort of metastatic BC patients [9]. To the best of our knowledge, there is only one study of 16 patients with HR+/HER2− metastatic BC [29], reporting the detection of PD-L1+ CTCs in the majority of CTC-positive patients as compared to only one third of CTC-positive patients described in our study. This discrepancy could be related to differences in the methods used for CTC enrichment, in the patient populations studied, and in the PD-L1 antibodies used. The E1L3N PD-L1 clone used in this report has been previously validated in several studies [32] and demonstrated the highest performance among different clones, for both cancer and immune cells, in triple-negative breast tumors [33].

Different clinical characteristics and outcomes have been described for patients with de novo metastatic BC compared to patients with recurrence after prior treatment for early disease [34,35]. De novo metastatic BC harbors distinct genomic profiles compared to recurrent disease, potentially indicating the presence of differential mechanisms of metastatic progression [36]. Our results suggest that de novo metastatic BC may present an increased potential for immune escape compared to recurrent disease or alternatively, prior systemic treatment for early BC may have selected CTC subsets bearing different immune escape properties. Nevertheless, these findings merit further investigation for understanding the biology of metastasis and envisioning novel therapeutic approaches for patients with de novo metastatic BC. 

The frequency of CD47^high^ and/or PD-L1^high^ CTCs significantly prevailed in triple-negative metastatic patients, implying that CD47 and PD-L1 immune checkpoints are preferentially involved in the immune escape of triple-negative BC. In line with our findings, CD47^high^CD68^high^ expression was more abundant in HR-negative BC tissue compared to luminal tissue [37], and also, increased PD-L1 expression rates were reported in triple-negative breast tumors [11,38]; however, this association is reported for the first time at the CTC level. Importantly, here, we demonstrate, for the first time, that CD47^high^ and/or PD-L1^high^ CTCs present significant prognostic implications, defining a group of metastatic patients presenting a higher risk of progression during first-line treatment. Moreover, the detection of these CTCs emerged as an independent predictor of an increased risk of relapse and death. The above results suggest that the expression of key regulators of innate and adaptive immunity on CTCs could serve as useful prognostic biomarkers in metastatic BC. In contrast, in early disease, CD47 and/or PD-L1 expression was not associated with patient outcomes, which could be related to the relatively small number of patients analyzed and the limited number of events. However, a large meta-analysis on the clinical relevance of PD-L1 expression in primary BC tumor tissue revealed that PD-L1 expression independently predicts for reduced OS [11]. In addition, tissue CD47 expression alone [39] or in combination with CD68 [37] was a poor prognostic indicator in primary BC. These observations reinforce the need for the further evaluation of CD47 and PD-L1 expression on CTCs in larger groups of early BC patients.

PD-L1 expression was more commonly detected on TILs compared to the respective tumor cells within BC tissue, in accordance with previous reports demonstrating differential PD-L1 expression rates among tumor cells and TILs in the tumor microenvironment [40,41,42]. Moreover, a positive correlation was shown among PD-L1-expressing TILs and tumor cells, suggesting that PD-L1 expression on tumor cells is related to immune infiltration [42,43]. On the contrary, in the blood, PD-L1 expression was more common on CTCs compared to PBMCs, corroborating previous observations in head and neck squamous cell carcinoma (HNSCC) [44]. In addition, the low concordance in PD-L1 positivity among CTCs and PBMCs indicates different mechanisms of PD-L1 regulation in the blood and tissue compartments [40]. Importantly, when peripheral blood and tumor tissue were compared, CTCs and primary tumor cells presented similar CD47 and PD-L1 expression rates; however, the positivity concordance was low. In contrast, PD-L1 expression was more frequently encountered on TILs in the tumor compared to PBMCs in peripheral blood. Collectively, the above observations suggest that evaluation of the systemic immune response in the blood in parallel to the local immune response within the tumor microenvironment, may provide a broader view of immune reactivity against the tumor [45]. 

The strengths of our study include the evaluation of well-defined cohorts of unselected patients with early, recurrent, and de novo metastatic BC, which allowed an investigation of the distribution and clinical relevance of immune checkpoint regulators on CTCs at different stages of disease progression. Furthermore, the evaluation of matched tumor and blood samples contributes to the limited existing data on the relative representation of immune molecules in peripheral blood and cancer tissue.

In the current study, the hematopoietic marker CD45 was not included in the immunofluorescence panel due to the limitation of our method in using up to three markers, and as a consequence, CTC detection was based on the detection of CK expression only [24,25,46,47,48]. Moreover, since CTC analysis is limited to rare events quantified at the single cell level, the detection of at least one positive or high-expressing CTC was used to define positivity or high expression, respectively, in contrast to tumor analysis, where the percentage of positive cells was evaluated. Nevertheless, different approaches have also been previously applied to characterize the expression of CD47 or PD-L1 on tumor tissue and CTCs [9,49,50,51]. Finally, the relatively small number of CTC-positive patients, as well the heterogeneity of the population studied, prevent firm conclusions from being drawn regarding the prognostic role of the different CTC subsets. 

To summarize, here, we show that CTCs expressing CD47 and/or PD-L1 are more frequently evident in de novo metastatic BC compared to early BC and that they independently predict for poor outcomes in metastatic patients treated with first-line therapy. These findings suggest a potential role of innate and adaptive immune evasion mechanisms in the metastatic capacity of CTCs and they highlight the significance of CTC analysis at the protein level. The differential distribution of CD47 and PD-L1 expression within the blood and tissue microenvironments suggests that the peripheral immune response merits further evaluation in parallel to the local response for better understanding the tumor immune reactivity. Investigations using modern morphologic, flow cytometric, and functional tests could significantly contribute to delineation of the clinical value of systemic and local immune responses in cancer patients [52]. Finally, CD47 and PD-L1 represent valid targets for the treatment of solid tumors, including BC [15,17,53], whereas preclinical evidence also indicates that their combined targeting is more effective compared to monotherapy [13,54]. Conceivably, CD47 and PD-L1 expression on CTCs merits further study as a non-invasive tool for the real-time monitoring of patients who could benefit most from these approaches.

## 4. Materials and Methods 

### 4.1. Study Design

The current study included patients with early (*n* = 100) and metastatic (*n* = 98) BC, who received adjuvant and first-line treatment, respectively, at the Department of Medical Oncology of the University General Hospital of Heraklion (Crete, Greece). Peripheral blood samples were obtained before the start of adjuvant or first-line treatment and cytospins of peripheral blood mononuclear cells (PBMCs) were prepared. Two slides per patient (total number: *n* = 396) were analyzed for CTC detection and characterization, according to CD47 and PD-L1 expression. The two markers were in parallel investigated in matched PBMCs from CTC-positive patients, as well as in matched primary (*n* = 26) or metastatic (*n* = 7) tumor tissue of CTC-positive patients with available tissue samples.

Clinical characteristics and follow-up information were prospectively collected. This study was conducted in accordance with the Declaration of Helsinki ethical guidelines and was approved by the Ethics and Scientific Committees of the University General Hospital of Heraklion (10751/6-9-2017), Crete, Greece. All patients gave their written informed consent to participate in the study.

### 4.2. Cell Culture

Cell lines were obtained from the American Type Culture Collection (ATCC). MCF-7 and MDA.MB.231 breast cancer cell lines were cultured in high glucose GlutaMAX™ Dulbecco’s Modified Eagle Medium (DMEM) (GIBCO-BRL Co, MD, USA), supplemented with 10% fetal bovine serum (FBS) (GIBCO-BRL) and 1% penicillin/streptomycin (GIBCO-BRL). MCF-7 cell culture medium was supplemented with 0.28% insulin. SKBR-3 cells were cultured in high-glucose GlutaMAX™ McCoys5A medium (GIBCO-BRL) supplemented with 10% FBS and 1% penicillin/streptomycin. Cells were maintained in a humidified atmosphere of 5% CO_2_–95% air at 37 °C and subcultivation was performed using 0.25% trypsin and 5 mM ethylenediaminetetraacetic acid (EDTA) (GIBCO-BRL). Following mycoplasma testing by the use of the MycoAlert^TM^ assay, cell cytospins were prepared in order to be included in the subsequent immunofluorescence (IF) stainings. 

### 4.3. Enrichment of CTCs in Blood Samples

Peripheral blood (10 mL) was obtained at the middle of vein puncture after the first 5 mL were discarded to avoid contamination with epithelial skin cells. PBMCs were isolated by Ficoll-Hypaque density gradient (d = 1077 g/mL) centrifugation at 650 g for 30 min. PBMCs were washed two times with phosphate-buffered saline (PBS) and aliquots of 500,000 cells were cyto-centrifuged at 500 g for 2 min on glass slides. Air-dried cytospins were stored at −80 °C until use.

### 4.4. Immunofluorescence (IF)

Triple IF staining for CK/CD47/PD-L1 was performed on cytospins prepared from BC cell lines or patients’ PBMCs. Cytospins were fixed with PBS/FA 3.7% for 15 min and permeabilized with PBS/Triton X-100 0.1% for 10 min, followed by 1h blocking with PBS/FBS 5% at room temperature (RT). Cytokeratins (CKs) were detected by two different Alexa Fluor 488-conjugated clones (mouse AE1/AE3 (1:100), Thermo Fisher Scientific, Waltham, Massachusetts, USA) and mouse C11 (1:200), Novus Biologicals, LLC, Centennial, Colorado, USA), after an overnight incubation at 4 °C. The primary antibodies, sheep anti-CD47 (R&D Systems, Minneapolis, USA, cat# AF4670) (1:100) and rabbit anti-PD-L1 (Clone E1L3N, Cell Signaling, Danvers, Massachusetts, USA, cat# 13684) (1:100), were incubated for 1 h, RT. The corresponding secondary antibodies, Dylight 550 anti-sheep (1:300) and Alexa Fluor 633 anti-rabbit (1:600), were incubated for 45 min, RT. Dapi antifade (Invitrogen) was finally added for identification of the cell nucleus.

### 4.5. Evaluation of CD47 and PD-L1 Expression in BC Cell Lines

Cytospins of SKBR-3, MCF-7, and MDA.MB.231 were triple stained for CK/CD47/PD-L1. For each cell line, one slide served as a triple-positive control (including all three primary and the corresponding secondary antibodies), whereas three slides served as negative controls, one for each marker (including the secondary immunoglobulin G (IgG) isotype antibody only and omitting the corresponding primary antibody) (Figure S1). A high expression of both CD47 and PD-L1 was evident in MDA.MB.231 cells only (Figure S1), which were consequently selected to serve as controls for patient samples.

CD47 and PD-L1 expression levels were first measured in MDA.MB.231 cells, using the Ariol microscopy system (Genetix, New Milton, UK). As described in our previous reports [25,47], intensity values represent the exposure time required for detection of the fluorescent signal. The intensity of each marker was measured among 1.000 MDA.MB.231 cells in the corresponding negative control and the lowest value represented the cut-off used to discriminate positive from negative expression. The intensity of each marker was then measured among 1.000 high-expressing MDA.MB.231 cells detected in the positive control and the highest value served as the cut-off to define high expression. Intermediate values positioned between the negative and high cut-offs were used to define low expression. 

CD47 and PD-L1 expression was then measured in SKBR-3 and MCF-7 cells and was accordingly characterized as negative, positive, high, or low, by using the previously defined cut-offs in the MDA.MB.231 control cells. 

### 4.6. Evaluation of CD47 and PD-L1 Expression in CTCs and PBMCs

A total of 1 × 10^6^ PBMCs (two slides) per patient (total number of slides: *n* = 396) were stained for CK/CD47/PD-L1 and analyzed using the Ariol microscopy system, as previously described [25,50,52]. The analysis was performed by three observers (P.G.T., A.M., and A.C.M.), who were blinded to each other’s findings and patients’ clinical data. The expression of CK was used to distinguish CTCs (CK-positive cells) and PBMCs (CK-negative cells).

MDA.MB.231 cell cytospins were included as controls in all IF stainings performed for patient samples. CD47 and PD-L1 expression was first measured in MDA.MB.231 cells (positive and negative controls) and then on single CTCs and PBMCs. As described above for the cell lines, CTCs and PBMCs were characterized as negative, positive, high, or low for CD47 and PD-L1 expression, by using the previously defined cut-offs in the MDA.MB.231 control cells.

The detection of at least one CTC with a positive or high expression was used to define the positivity or high expression of CTCs, as previously described [25,46,47,48]. The two markers were also characterized among 1.000 PBMCs in randomly selected microscopy vision fields and the detection of ≥1% PBMCs with a positive or high expression was used to define the positivity or high expression of PBMCs.

The subcellular localization of CD47 and PD-L1 was further analyzed by using Confocal Laser Scanning Microscopy (CLSM). Membranous CD47 staining was evaluated on CTCs and PBMCs. Membranous PD-L1 staining and membranous, cytoplasmic, or punctuate staining was defined as positive in CTCs and PBMCs, respectively.

### 4.7. Immunohistochemistry (IHC)

Three-micron-thick formalin-fixed paraffin-embedded tissue sections were deparaffinized in xylene and rehydrated in a graded series of ethanols, and antigen retrieval was induced by heating the sections in 1 mM EDTA buffer, at pH 8.0, for 45 min, using a steam pot. Consequently, endogenous peroxidase and nonspecific protein-binding site blocking was implemented and sections were stained using the CD47 sheep polyclonal antibody (R&D Systems, Minneapolis, MN, USA, cat# AF4670), and with PD-L1 rabbit monoclonal clone E1L3N (Clone E1L3N, Cell Signaling, Danvers, MA, USA, cat# 13684), both at 1:200 for 1 h at room temperature. Immunodetection was performed using Cell and Tissue Staining Kit HRP-DAB CTS019 (R&D Systems) for CD47 and UltraVision Quanto Detection System HRP Polymer DAB (Thermo Fisher Scientific, Waltham, MA, USA) for PD-L1, according to each manufacturer’s instructions. Ultimately, slides were rinsed with distilled water for 5 min and then counterstained with haematoxylin. Positive (placenta for CD47 and tonsil for PD-L1) and negative controls were used.

### 4.8. Evaluation of CD47 and PD-L1 Expression on Tissue Samples

The immunohistochemical expression of CD47 and PD-L1 was scored by two experienced pathologists (A.V.K. and E.L.), blinded to patients’ clinical data. Discordant scores were reviewed with a dual-head microscope until a consensus was reached. CD47 expression was semi-quantitatively scored by assessing both the intensity of staining (graded as: 0: no-staining; 1: weak; 2: moderate; or 3: strong) and the percentage of positive tumor cells as 0 (none), 1 (1–10%), 2 (11–50%), 3 (51–80%), or 4 (>80%). Multiplication of the intensity and percentage resulted in an immunoreactivity score, ranging from 0 to 12 for each individual case, and scores ≥1 and ≥7 were used to define CD47 positivity and high CD47 expression, respectively, as previously described [9]. For PD-L1 expression, the percentage of stained tumor cells (membranous staining) and TILs (cytoplasmic, membranous, or punctuate staining) was separately evaluated and enumerated from 0–100% after a thorough examination of the entire representative viable tumor area included in the slide. For both cell subsets, PD-L1-positivity and high PD-L1 expression were determined by using the cut-off values of ≥1% and ≥5% of stained cells at any intensity, respectively.

### 4.9. Statistical Analysis

Fisher’s exact test was used to compare the incidence of distinct cell subsets and to investigate possible correlations with patient and disease characteristics. Kaplan–Meier analysis was used to estimate survival curves. The disease-free interval (DFI) was calculated from the start of adjuvant treatment until the day of documentation of disease progression. Progression-free survival (PFS) was calculated from the start of first-line treatment until disease progression or death from any cause. Overall Survival (OS) was calculated from the treatment initiation until death from any cause. Univariate Cox regression analysis, along with a multivariate Cox proportional hazards regression model, were performed to investigate the associations between different parameters and the risk for relapse and death. Statistical analyses were performed using IBM SPSS Statistics version 20. P values were calculated by two-sided tests and were considered statistically significant at the 0.05 level.

## 5. Conclusions

The results presented here indicate that CTCs expressing CD47 and/or PD-L1 prevail in de novo metastatic BC compared to early BC and have an independent prognostic significance in metastatic patients treated with first-line therapy. A differential distribution is demonstrated for CD47 and PD-L1 expression on tumor and immune cells, among peripheral blood and tumor tissue microenvironments. The above findings suggest that innate and adaptive immune evasion mechanisms operate on CTCs and could be involved in their metastatic potential. The findings also imply that the clinical value of the parallel assessment of peripheral and local immune response merits further evaluation in BC.

## Figures and Tables

**Table 1:** Patient and disease characteristics of patients with early and metastatic breast cancer (BC).

Early BC Patients (*n* = 100)	*n* (%)	Metastatic BC Patients (*n* = 98)	*n* (%)
Age, years		Age, yrs	
Median (range)	55 (32–81)	Median (range)	59 (29–84)
PS		PS	
0–1	97 (97)	0–1	92 (93.9)
2	3 (3)	2	3 (3.1)
		Unknown	3 (3.1)
Histology		Histology	
Ductal	83 (83)	Ductal	80 (81.6)
Lobular	11 (11)	Lobular	10 (10.2)
Mixed	2 (2)	Mixed	4 (4.1)
Unknown	4 (4)	Unknown	4 (4.1)
Grade		Stage at diagnosis	
I–II	44 (44)	I–III	71 (70.4)
III	43 (43)	IV	27 (27.6)
Unknown	13 (13)		
Stage		Subtype	
I	22 (22)	HR+/HER2−	63 (64.3)
ΙΙ	60 (60)	HER2+	22 (22.5)
IΙΙ	14 (14)	Triple-negative	12 (12.2)
Unknown	4 (4)	Unknown	1 (1)
Subtype		Prior adjuvant treatment	
HR+/HER2−	71 (71)	Yes	66 (67.3)
HER2+	17 (17)	No	28 (28.6)
Triple-negative	10 (10)	Unknown	4 (4.1)
Unknown	2 (2)		
Adjuvant treatment ^a^ Chemotherapy Hormone therapy	97 (97) 77 (77)	Organs affected Breast Bones CNS Lung Liver LNs Other	26 (26.5) 36 (36.7) 10 (10.2) 41 (41.8) 34 (34.7) 39 (39.8) 9 (9.2)
		Disease sites 1–2 >2 Unknown	61 (62.2) 33 (33.7) 4 (4.1)
		First line treatment ^a^ Chemotherapy Hormone therapy Unknown	88 (89.8) 9 (9.2) 1 (1)
		Response to treatment PR SD PD NE	41 (41.8) 31 (31.6) 18 (18.4) 8 (8.2)

^a^ Patients with HER2-positive disease received trastuzumab; NE; non-evaluable.

**Table 2:** Incidence of CD47 and PD-L1 expression on CTCs of patients with metastatic BC (*n* = 97) according to the BC subtype.

CTC Populations	CTC Detection According to BC Subtype (% of Patients)			*p* Value
	Triple-Negative	HR+/HER2−	HER2+	
Total CTCs	50	20.6	13.6	0.053
CD47+	50	17.5	13.6	0.045 *
CD47^high^	25	11.1	0	0.049 *
PD-L1+	25	4.8	0	0.025 *
PD-L1^high^	16.7	4.8	0	0.097
CD47+and/orPD-L1+	50	17.5	13.6	0.045 *
CD47^high^and/orPD-L1^high^	33.3	11.1	0	0.015 *

* Statistical significance at the *p* < 0.05 level; Two-sided Fisher’s exact test.

**Table 3:** Incidence of CD47 and PD-L1 expression on CTCs of patients with metastatic BC (*n* = 90) according to best response at first evaluation of treatment.

CTC Populations	CTC Detection According to Response to Treatment (% of Patients)		*p* Value
	PD	PR/SD	
Total CTCs	44.4	16.7	0.011 *
CD47+	38.9	15.3	0.025 *
CD47^high^	22.2	5.6	0.026 *
PD-L1+	11.1	2.8	0.177
PD-L1^high^	11.1	1.4	0.101
CD47+and/orPD-L1+	38.9	15.3	0.025 *
CD47^high^and/orPD-L1^high^	27.8	5.6	0.005 *

* Statistical significance at the *p* < 0.05 level; Two-sided Fisher’s exact test.

**Table 4:** Univariate and multivariate Cox-regression analysis for PFS and OS among patients with metastatic BC.

Cox Regression Analysis	Progression-Free Survival (PFS)				Overall Survival (OS)			
	Univariate		Multivariate		Univariate		Multivariate	
Covariates	HR (95% CI)	*p* Value	HR (95% CI)	*p* Value	HR (95% CI)	*p* Value	HR (95% CI)	*p* Value
Age (>59)	0.811 (0.512–1.283)	0.370			0.677 (0.409–1.120)	0.129		
Performance status (0–1)	0.431 (0.133–1.402)	0.162			0.363 (0.112–1.178)	0.091		
Recurrent disease	1.575 (0.944–2.626)	0.082			1.837 (1.013–3.331)	0.045 *	4.072 (1.970–8.418)	0.000 *
*Molecular subtype of tumor*								
HR-positive	0.910 (0.522–1.587)	0.740			0.918 (0.504–1.675)	0.781		
HER2-positive	1.607 (0.923–2.798)	0.094			1.794 (0.951–3.385)	0.071		
Triple-negative	1.559 (0.796–3.054)	0.195			1.401 (0.688–2.850)	0.353		
No of organs affected (>2)	1.453 (0.914–2.310)	0.115			2.084 (1.255–3.461)	0.005 *	3.456 (1.897–6.294)	0.000 *
*Metastatic sites*								
Liver	1.613 (1.017–2.560)	0.042 *	1.816 (1.126–2.930)	0.014 *	2.002 (1.195–3.353)	0.008 *	1.999 (1.142–3.499)	0.015 *
Lung	0.995 (0.631–1.568)	0.982			0.825 (0.497–1.368)	0.455		
Bones	1.287 (0.809–2.049)	0.287			1.513 (0.911–2.514)	0.110		
Lymph nodes	0.825 (0.524–1.297)	0.404			0.688 (0.418–1.135)	0.143		
CNS	0.872 (0.434–1.751)	0.700			0.901 (0.427–1.901)	0.785		
Skin	0.841 (0.364–1.943)	0.685			0.716 (0.308–1.666)	0.439		
Total CTCs	1.866 (1.100–3.168)	0.021 *			1.558 (0.880–2.758)	0.128		
*CD47^high^* CTCs	2.192 (1.079–4.452)	0.030 *			1.433 (0.680–3.018)	0.344		
*PD-L1^high^* CTCs	2.977 (1.058–8.375)	0.039 *			2.793 (0.986–7.914)	0.053		
*CD47^high^and/orPD-L1^high^* CTCs	2.373 (1.203–4.682)	0.013 *	2.719 (1.302–5.677)	0.008 *	1.610 (0.792–3.272)	0.189	2.398 (1.071–5.371)	0.034 *

* Statistical significance at the *p* < 0.05 level.

**Table 5:** Comparison of the PD-L1 status between tumor cells and immune cells within peripheral blood and primary tumors.

PD-L1 Distribution among Tumor and Immune Cells	Peripheral Blood Patients (%)			Primary Tumor Patients (%)		
	CTCs	PBMCs	Positivity Concordance	Tumor Cells	TILs	Positivity Concordance
PD-L1 expression	27.8	22.2	11.1	34.6	65.4	34.6
PD-L1^high^ expression	19.4	5.6	2.8	11.5	42.3	11.5

PD-L1 expression on tumor and immune cells was investigated in blood samples (no of patients: *n* = 36) and primary tissue samples (no of patients: *n* = 26).

**Table 6:** Comparative analysis of CD47 and PD-L1 expression among the primary tumor tissue and peripheral blood.

**CD47 in Tumor cells**	**CD47 Expression** patients (%)				**CD47^high^ Expression** patients (%)	
	Primary Tumor	CTCs	Positivity Concordance	Primary Tumor	CTCs	Positivity Concordance
All patients	88.5	84.6	76.9	19.2	53.8	11.5
Early	80	70	60	20	60	20
Metastatic	93.8	93.8	87.5	18.8	50	6.2
**PD-L1 in Tumor cells**	**PD-L1 expression** patients (%)				**PD-L1^high^ expression** patients (%)	
	Primary tumor	CTCs	Positivity concordance	Primary tumor	CTCs	Positivity concordance
All patients	34.6	30.8	7.7	11.5	23.1	3.8
Early	30	30	0	20	20	0
Metastatic	37.5	31.2	12.5	6.2	25	6.2
**PD-L1 in Immune cells**	**PD-L1 expression** patients (%)				**PD-L1^high^ expression** patients (%)	
	TILs	PBMCs	Positivity concordance	TILs	PBMCs	Positivity concordance
All patients	64	20	16	44	8	4
Early	60	40	30	40	10	0
Metastatic	66.7	6.7	6.7	46.7	6.7	6.7

CD47 and PD-L1 expression on tumor cells and immune cells was in parallel investigated in matched primary tumor tissue and blood samples of patients with BC (total patients: *n* = 26; early setting: *n* = 10; metastatic setting: *n* = 16).

## Supplementary Materials

The following are available online at https://www.mdpi.com/2072-6694/12/2/376/s1: Figure S1: CD47 and PD-L1 expression in BC cell lines; Figure S2: Kaplan–Meier curve for PFS of patients with metastatic BC, according to the combined versus single CD47^high^ or PD-L1^high^ expression on CTCs; Table S1: Comparative analysis of CD47 and PD-L1 expression in matched primary tissue, metastatic tissue, and peripheral blood.
